# Omega-3 polyunsaturated fatty acids do not fluidify bilayers in the liquid-crystalline state

**DOI:** 10.1038/s41598-018-34264-3

**Published:** 2018-11-02

**Authors:** Augusta De Santis, Yaiza Varela, Jesús Sot, Gerardino D’Errico, Félix M. Goñi, Alicia Alonso

**Affiliations:** 1 Instituto Biofisika (CSIC, UPV/EHU), B. Sarriena s/n, 48940 Leioa, Spain; 2 Department of Chemical Sciences, University of Naples “Federico II”, Complesso di Monte S. Angelo, Via Cinthia, I-80126 Naples, Italy; 3 CSGI, Consorzio Interuniversitario per lo Sviluppo dei Sistemi a Grande Interfase, Via della Lastruccia 3, Sesto Fiorentino, 50019 Florence, Italy; 4 Departamento de Bioquímica, Universidad del País Vasco, 48940 Leioa, Spain

## Abstract

This work reports on the effects of two omega-3 fatty acids, namely docosahexaenoic (C22:6^4,7,10,13,16,19^) acid (DHA), and eicosapentaenoic (C20:5^5,8,11,14,17^) acid (EPA), with oleic (C18:1^9^) acid (OA) as a control, on the gel-liquid crystalline phase transition of dipalmitoyl phosphatidylcholine (DPPC). Mainly differential scanning calorimetry has been used, together with Laurdan fluorescence, and confocal fluorescence microscopy. All three fatty acids DHA, EPA and OA exhibited fluidifying properties when added to the DPPC bilayers, decreasing the main transition temperature. DHA and EPA were somewhat more effective than OA in this respect, but the effects of all three were of the same order of magnitude, thus the long-chain omega-3 fatty acids failed to exhibit any peculiar fluidifying potency. The same was true when the omega-3 fatty acids were esterified in the sn-2 position of a phosphatidylcholine. Moreover the omega-3 fatty acids had very small or no effects on the fluidity of bilayers in the liquid-crystalline, or fluid disordered state (egg phosphatidylcholine and others), or in the fluid ordered state (phospholipid: cholesterol mixtures). The hypothesis that some physiological effects of long-chain omega-3 fatty acids could be related to their special fluidifying properties is not supported by these data.

## Introduction

Omega-3 fatty acids are polyunsaturated fatty acids (PUFAs) with a double bond (C=C) at the third carbon atom from the end of the carbon chain. The dietary intake of omega-3 fatty acids such as docosahexaenoic (DHA), or C22:6^4,7,10,13,16,19^ and eicosapentaenoic (EPA), or C20:5^5,8,11,14,17^ has been associated with the prevention of cancer and heart diseases, although these are debated issues^1,2^. These fatty acids are deemed essential in neurological and brain development^3,4^. Among other hypotheses, mainly based on their radical scavenging and anti-inflammatory action^5^, it has been proposed that omega-3 fatty acids, once incorporated into phospholipids, could alter the structure and functionality of biological membranes^6^.

Previous studies on the effects of omega-3 fatty acids on membranes have been carried out mostly with the fatty acids incorporated into phospholipids^7–9^. Nevertheless the role of free fatty acids should also be taken into account; several membrane phenomena, e.g. vesicle cycling, involve the interplay between membrane lipids and the enzyme phospholipase A_2_ (PLA2)^10^. The PLA2 family consists of a group of enzymes that hydrolyse the ester bond at the *sn*-2 position in glycerophospholipids, inducing the release of a free fatty acid and a lysophospholipid. In neural cells, PLA2 was shown to control the metabolic transformation of phospholipid molecules containing polyunsaturated fatty acids (PUFAs)^11^. In addition Georgieva *et al*.^12^, using raft-like lipid mixtures, demonstrated that line tension and elastic properties govern budding formation after the addition of PLA2, which explains why DHA- containing phosphatidylcholines, but not oleic acid (OA)- containing phosphatidylcholines, are able to exhibit liquid-ordered (L_o_) domain budding.

Relatively little is known about the interaction of free fatty acids with phospholipid bilayers. Ehringer *et al*.^9^ compared the properties of DHA and of its metabolic precursor linolenic acid (C18:3^9,12,15^) both in phospholipid form and as free fatty acids. These authors, on the basis of fluorescence polarization studies, observed that incorporation of either linolenic acid or of DHA broadened and depressed the temperature of the phase transition, while they had almost no effect on the fluidity of the liquid-crystalline phase. They also found that DHA was more potent than linolenic in facilitating membrane fusion and permeability. They concluded that there is a clear distinction between the effects of DHA and linolenic acid in membranes^9^. Valentine and Valentine^13^ reviewed the properties of omega-3 fatty acids in cellular membranes and concluded that DHA and EPA contribute “hyperfluidizing” properties to the membrane, which distinguish those highly unsaturated chains from the monounsaturated ones.

The present contribution intended to expand our understanding of the effects of omega-3 polyunsaturated fatty acids, namely DHA and EPA, with OA as a control, on the fluidity of dipalmitoyl phosphatidylcholine (DPPC) membranes. The study was carried out using differential scanning microcalorimetry (DSC); in addition, generalized polarization of Laurdan was examined in order to obtain further information on the fluidity of membranes, and confocal microscopy of giant unilamellar vesicles (GUVs) containing DHA was performed to explore the existence of microdomains. Our data suggest that there is not a qualitative difference between mono- and polyunsaturated fatty acids in their fluidifying effects, rather a difference of degree is observed.

## Results

### Differential scanning calorimetry

**Figure 1:**
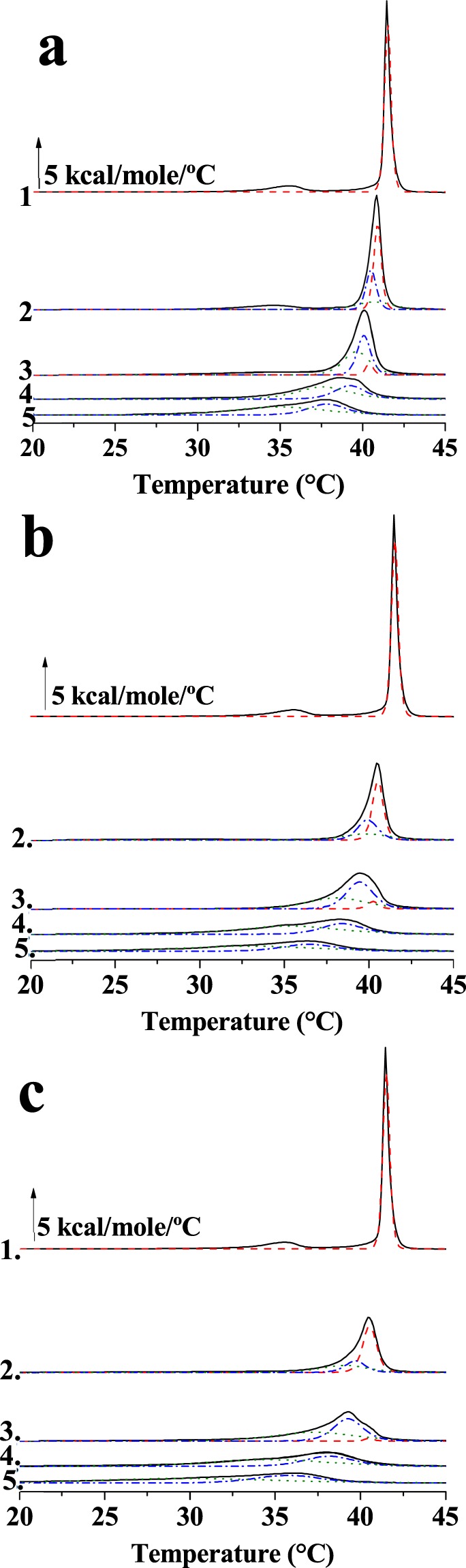
Representative thermograms from differential scanning calorimetry experiments for the mixtures: (1) pure DPPC, or DPPC containing 5 mol% (2), 10 mol% (3), 20 mol% (4), or 30 mol% (5) of oleic acid (**a**), eicosapentaenoic acid (**b**) and docosahexaenoic acid (**c**). Fittings of the thermograms to 2–3 gaussian components are shown in dashed lines.

To explore the interaction between DPPC and the three free fatty acids OA, EPA, and DHA, a detailed calorimetric study was performed to characterise the changes in the DPPC gel-fluid lamellar phase transition. DPPC was selected as the main bilayer component because of its very narrow (i.e. cooperative) thermal transition endotherm, which allows an easy detection of subtle changes in the transition parameters. Representative thermograms for the mixtures containing 0%, 5%, 10%, 20% and 30% of each fatty acid are shown in Fig. 1.

Pure DPPC displays a cooperative rippled (Pβ’)-to-liquid crystalline (L_α_) phase transition, marked by an endothermic signal centred at 41.4 °C. This so-called main transition is preceded by a smaller pre-transition endothermic peak at 35.6 °C, associated to the gel phase (Lβ)-to-(Pβ’) phase change^14^. Treatment with unsaturated fatty acids downshifted the phase transition temperature of DPPC in a dose-dependent manner. Even a small concentration, e.g. 5 mol%, of either monounsaturated or polyunsaturated fatty acids induced clear changes in the DPPC thermogram. OA caused the downshift of the main and pre-transition peaks, the latter still visible. With the polyunsaturated fatty acids however the pre-transition endotherm was no longer detected. All three free fatty acids caused in addition a broadening of the DPPC main transition endotherm, an effect that increased in the order OA < EPA < DHA. The increased width appeared to be due to a shoulder at the lower-temperature side of the endotherm, more marked for DHA. Increasing the amount of each free fatty acid, the sharp peak associated with the DPPC main transition, became progressively more broad and asymmetric (Fig. 1).

**Figure 2:**
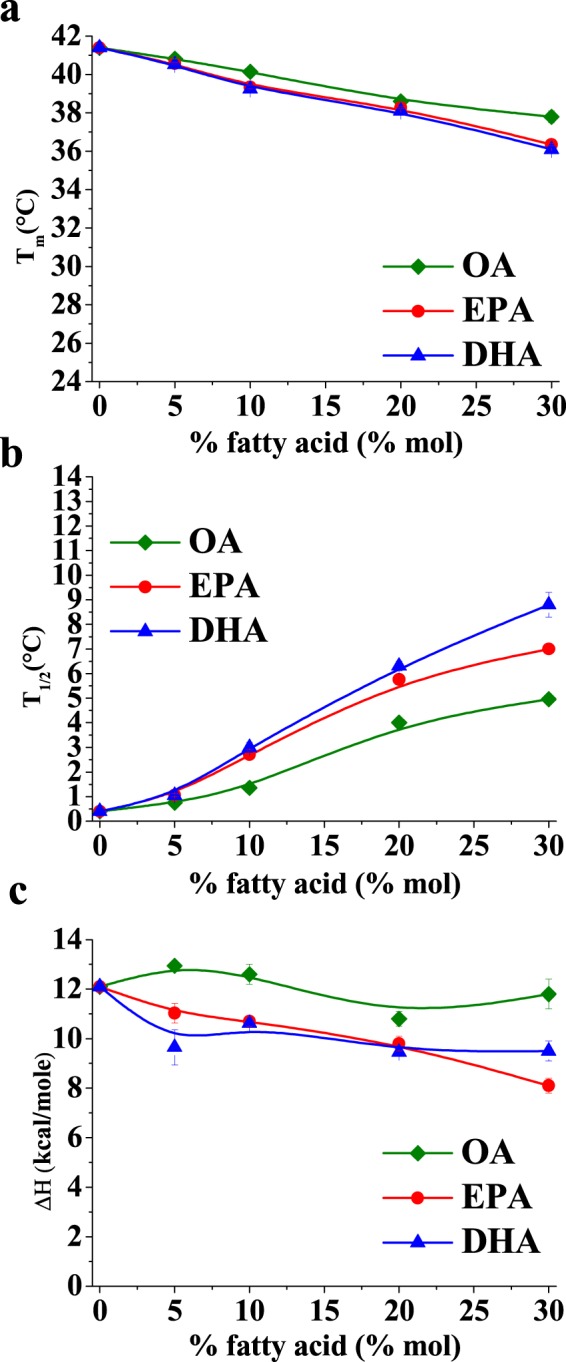
Average values of (**a**) main transition temperature (T_m_), (**b**) width at half-height (T_1/2_) and (**c**) enthalpy change (ΔH). Each value is the average of calorimetry experiments performed on two independent liposome preparations. Average ± S.D. Sometimes the error bars are smaller than the symbols.

Transition temperatures, enthalpies, and widths at half-height of the mixtures reported in Fig. 1 were determined using the software ORIGIN (Microcal) provided with the calorimeter. Concerning the transition temperatures, Fig. 2a shows that Tm decreased in a dose-dependent way for all three fatty acids, suggesting that these molecules tend to fluidify the DPPC membrane. Widths at half-height (T_1/2_) (Fig. 2b) describe the cooperativity of the transition: the higher the T_1/2_ value the less cooperative is the process; increasing the amount of free fatty acids also increased T_1/2_, thus the melting process became less cooperative. Cooperativity decreased with free fatty acids in the order OA < EP0041 ≈ DHA. ΔH variations (Fig. 2c) also indicate that less heat was necessary to obtain the (Pβ’)-to-liquid crystalline (L_α_) phase transition in the presence of polyunsaturated fatty acids, in agreement with their fluidifying effect.

For further analysis of the calorimetric results, the thermograms in Fig. 1 were decomposed into their component endotherms using the software Origin 7.0 (MicroCal). Thermograms were individually fitted to the smallest number of Gaussian curves compatible with a statistically convergent solution with an R^2^ correlation coefficient >0.99; the best fittings are plotted as dotted lines in Fig. 1. Endotherm components are drawn in different colours for the sake of clarity.

**Figure 3:**
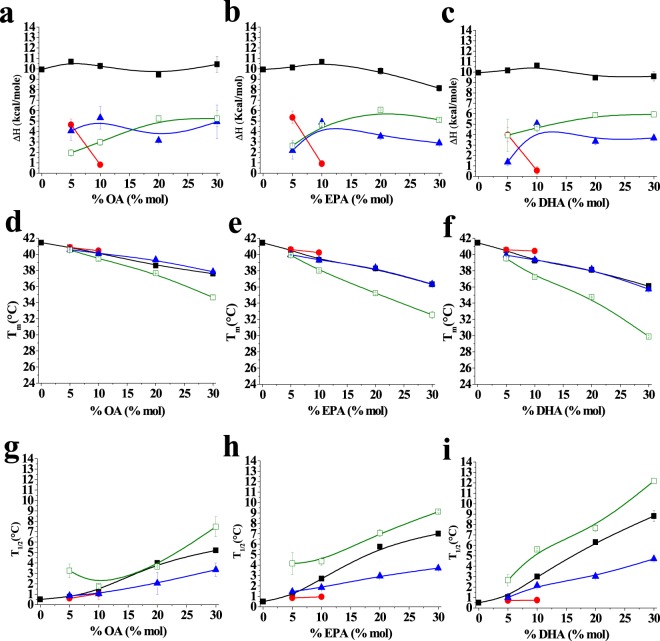
(**a**–**c**) Enthalpy change (ΔH), (**d**–**f**) transition temperature (T_m_), and (**g**–**i**) width at half-height (T_1/2_) mean values for each fitting component in the different mixtures. (**a**,**d** and **g**) show oleic acid containing mixtures; (**b**,**e** and **h**) show eicosapentaenoic acid containing mixtures and (**c**,**f** and **i**) show docosahexaenoic acid containing mixtures. Average ± S.D. of two independent liposome preparations. Sometimes the error bars are smaller than the symbols.

Two or three components (excluding the pre-transition) were sufficient to characterize the thermotropic transitions of the lipid mixtures containing free fatty acids (thermograms b-e in Fig. 1). To quantitatively analyse the behaviour of those components, in order to identify their origins, the transition temperature, the width-at-half-height (T_1/2_) and ΔH of each component endotherm were measured and are plotted in Fig. 3. For all the parameters the overall values are shown in black while values corresponding to the three components, detected by the fitting procedures, appear in different colours: red, blue or green.

In all the investigated samples, the component indicated in red disappeared upon addition of 10% OA, or EPA, or DHA. Such component exhibited the same T_m_ and T_1/2_ of the main transition of pure DPPC and those values remained virtually constant until the signal disappeared; thus, the ‘red’ component was ascribed to pure DPPC. Moreover the components reported in blue and green (respectively higher and lower T_m_) appeared as the fatty acids were added and varied in parallel with the increase in fatty acid molar fraction. For both of them the transition temperatures decreased as a function of free fatty acid concentration, indicating a fluidification of lipid system, but the effect was higher for the green one. Also their contributions to the cooperativity of the process (as indicated by the widths-at-half-height) were different, higher for the green component. The enthalpy changes of those two components (Fig. 3) were similar, and they exhibited little change with increasing free fatty acid concentrations. The fact that the properties of these two components varied monotonically with unsaturated fatty acid concentration suggests that both of them are qualitatively similar, i.e. both appear to arise from DPPC/free fatty acid mixtures, but differ in the proportion of free fatty acid, the green, low T_m_ component containing a higher proportion of the fatty acid. Note that DSC data do not allow us to distinguish between small and large domains, the coexistence of fatty-acid-rich and -poor mixtures can occur, as far as calorimetry is concerned, between micrometer- or nanometer-sized domains.

In summary, the DSC data strongly argue in favour of a non-ideal mixing of free fatty acids and saturated phospholipids in bilayers. The effects of OA, EPA and DHA increase regularly in this order, without a large discontinuity between the mono- and the poly-unsaturated species, suggesting that polyunsaturated chains do not possess any intrinsically peculiar property, at least from the point of view of their interaction with saturated phosphatidylcholines.

### Laurdan fluorescence emission

**Figure 4:**
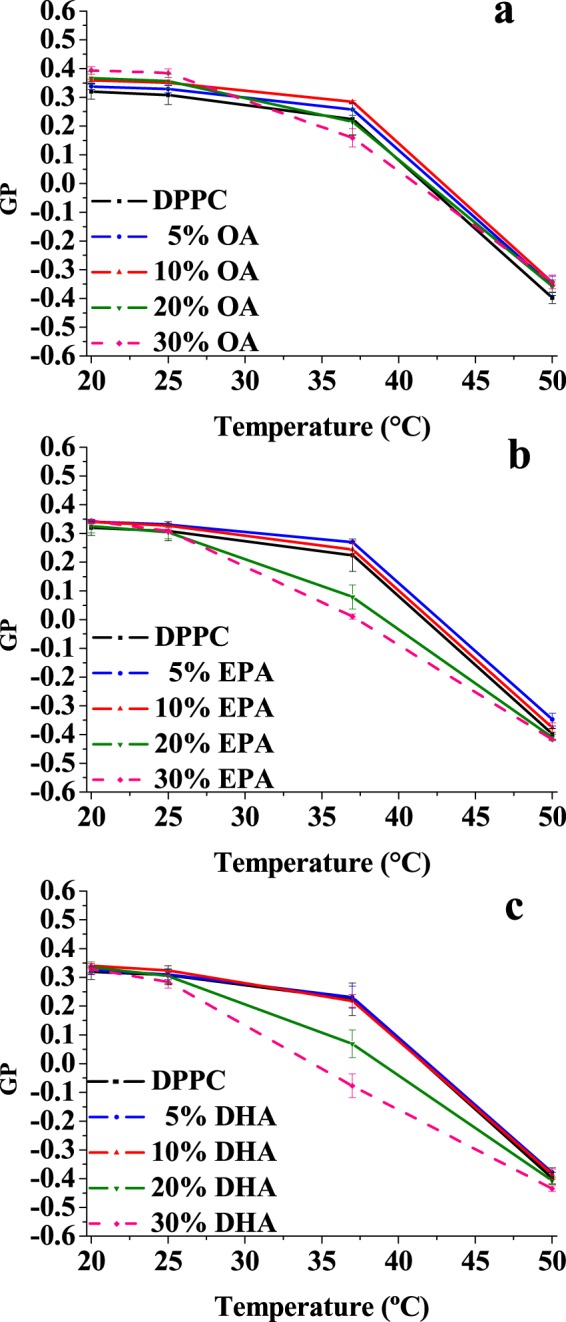
Mean Laurdan GP values of DPPC alone (black squares) and mixtures containing 5 mol%, 10 mol%, 20 mol% or 30 mol% of oleic acid (**a**), eicosapentaenoic acid (**b**), docosahexaenoic acid (**c**) at different temperatures. Average values ± S.D. of 3 independent liposome preparations.

Laurdan is a well-known fluorescence probe that reports on the lipid-water interface in lipid bilayers. The so-called generalised polarisation (GP) is a parameter derived from the Laurdan emission spectrum, whose change reflects faithfully the change in fluidity during the gel-fluid transition of phospholipids^15^. In order to examine from a different point of view the effect of free fatty acids on the packing of phospholipids in bilayers, we analysed Laurdan emission behaviour in simple two-component lipid mixtures consisting of DPPC with OA, EPA or DHA. The results are shown in Fig. 4 for the various free fatty acids, at different concentrations in the DPPC bilayer, and at different temperatures.

Addition of monounsaturated OA, at least below 30 mol%, causes little change of GP of Laurdan at all investigated temperatures; 30 mol% OA lowered GP slightly at 37 °C as compared to pure DPPC (Fig. 4a). This effect is more clearly seen with EPA and DHA, already at 20 mol% (Fig. 4b,c).

**Figure 5:**
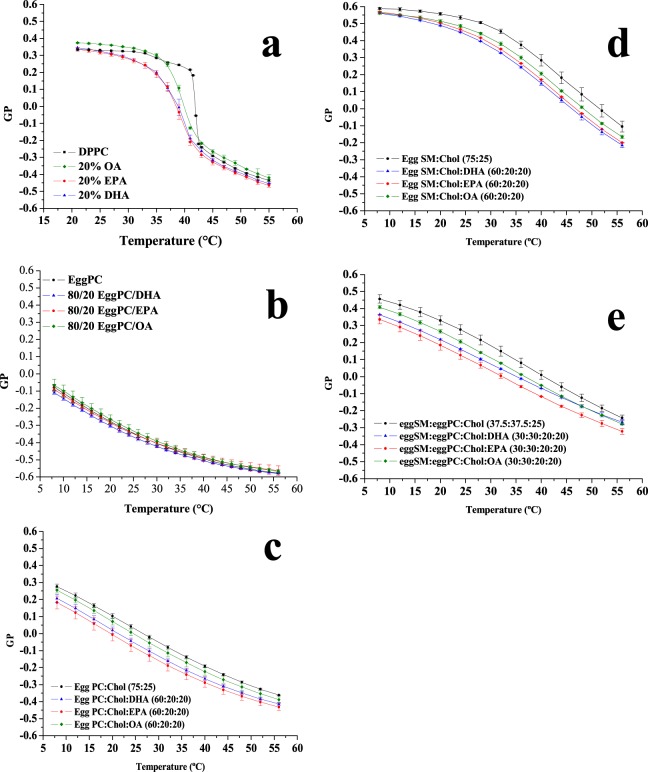
Temperature scans of Laurdan GP value in the absence and presence of 20 mol% of each free fatty acid with (**a**) DPPC, (**b**) egg PC, (**c**) egg PC/cholesterol (75:25 mol ratio), (**d**) egg SM/cholesterol (75:25 mol ratio), or (**e**) egg SM/egg PC/cholesterol (37.5:37.5:25 mol ratio). Average ± S.D. of 2–3 independent liposome preparations. Sometimes the error bars are smaller than the symbols.

A detailed temperature scan of Laurdan GP values was performed with free fatty acids at 20 mol% (Fig. 5a). From the inspection of Fig. 5a, the pre- and main transitions of pure DPPC, reported in black, are clearly seen, confirming that Laurdan GP is an efficient reporter to investigate the phase transitions in lipid mixtures. The presence of OA, EPA or DHA suppresses or blurs the pre-transition, induces a shift of the main transition to lower temperatures, and widens the transition temperature range. Under these conditions OA appears to be less potent than the other two fatty acids, while EPA and DHA effects are very similar to each other. All this is in agreement, at least semi-quantitatively, with the DSC results. The fluorescence observations are compatible with the Laurdan label residing in a more polar environment when polyunsaturated fatty acids are added to the lipid mixtures, suggesting that the lipid headgroups are less tightly packed, and more water molecules can access Laurdan.

Both DSC and Laurdan results suggest little effects of the free fatty acids on phospholipid bilayers in the fluid, or liquid-crystalline form. Furthermore Laurdan, but not DSC, can be conveniently used to assess the fatty acid effects on a fluid bilayer, such as one composed of pure egg PC, over a range of temperatures. This was tested, and the results are shown in Fig. 5b. Under these conditions, Laurdan GP did not allow the observation of any effects caused by the unsaturated free fatty acids.

To obtain information that could be more relevant to the situation in the cell membranes, and particularly in the plasma membranes, some experiments were performed on bilayers consisting of egg PC:cholesterol (75:25 mol ratio), egg SM:cholesterol (75:25 mol ratio), or egg SM:egg PC:cholesterol (37.5:37.5:25 mol ratio (Fig. 5c–e). Laurdan GP values of egg PC:cholesterol bilayers (Fig. 5c) were higher at all temperatures than the corresponding values for pure PC. The higher GP occurs as a result of the liquid ordered state of egg PC:cholesterol (75:25 mol ratio) bilayers. Perhaps because of this higher initial order, the unsaturated fatty acids do show in this case a small disordering effect, increasing in the order OA < EPA < DHA. However the difference in potency between the mono- and the poly-unsaturated fatty acids is also small, thus the data do not support the claim of a particular fluidifying potency of the omega-3 fatty acids. Rather the results are very similar to those found with DPPC bilayers (Figs 1 and 5a). Including SM in our studies was important because this lipid occurs at particularly high concentrations in the myelin sheaths of nerve cells, and omega-3 fatty acids are deemed essential in neurological and brain development. SM:Chol mixtures give rise to a particularly ordered fluid state^16^ as indicated by the very high GP values in Fig. 5d,e. Again the unsaturated fatty acids cause a slight disordering effect, but the membranes remain in all cases in the liquid ordered phase, with GP values well above those of the liquid disordered phase, represented in our case by the pure egg PC (Fig. 5a), and the effects of DHA or EPA are not clearly more intense than those of OA.

### Confocal microscopy of GUVs

**Figure 6:**
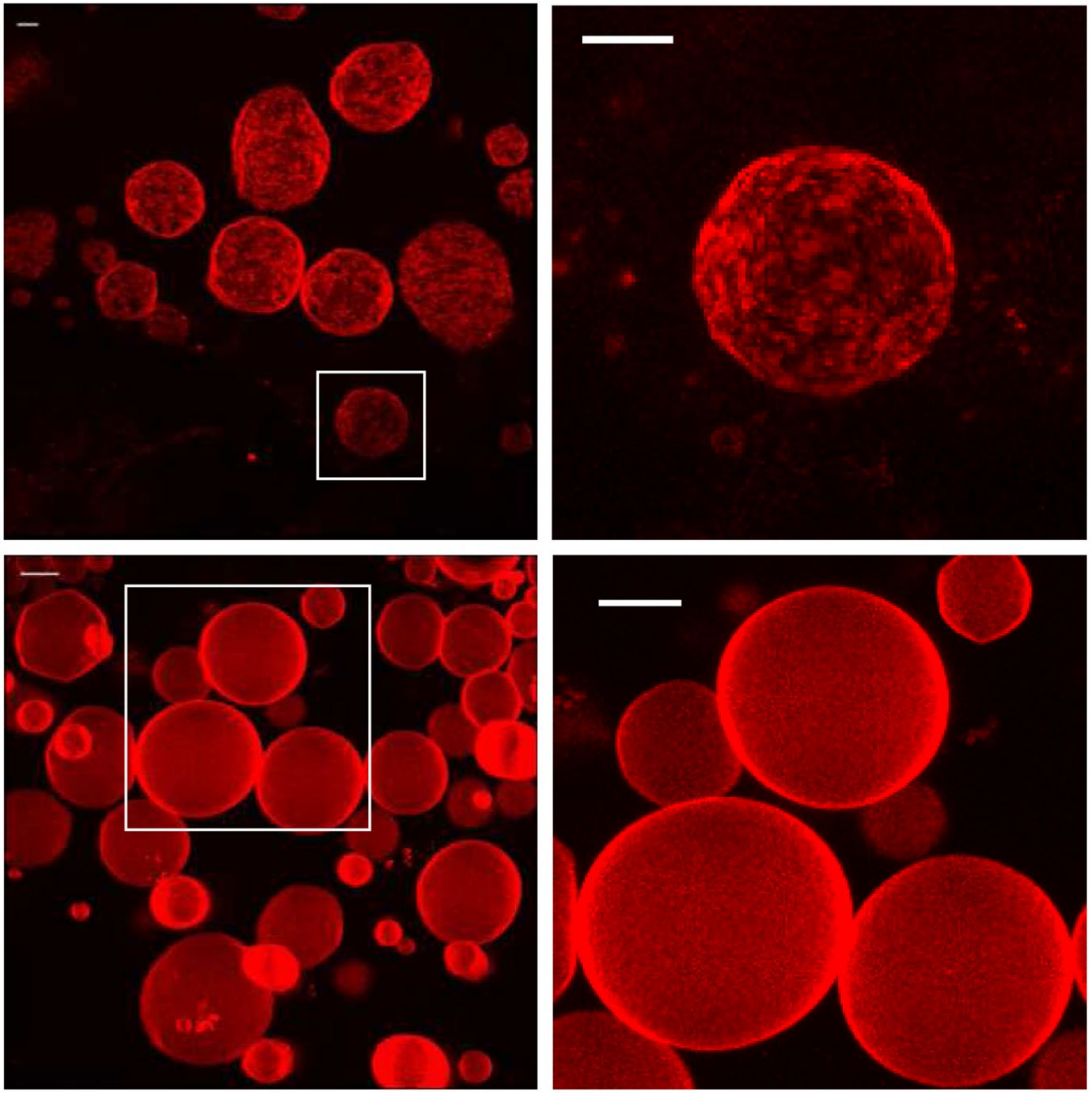
Representative Giant Unilamellar Vesicle images, labeled with Rho-PE, a probe that is known to partition preferentially into the more disordered phases. Top: DPPC. Bottom: 20% DHA + 80% DPPC (mol%). Observations at room temperature. Scale bar: 10 μm.

Rho-PE-stained GUVs of DPPC and DPPC/DHA (70:30 mole ratio) were prepared following an electroformation protocol, and observed by confocal microscopy after cooling down to room temperature. Rho-PE partitions into the more disordered phases^17^. In Fig. 6 (top) the images corresponding to pure DPPC GUVs are reported. The vesicles showed a rough, heterogeneous surface, probably due to the coexistence of gel and sub-gel (crystalline) phases, which occurs when fluid DPPC samples are annealed to room temperature. However when 30 mol% DHA was present in the lipid mixtures (Fig. 6, bottom) the vesicles looked smoother, as they do when existing in the liquid crystalline phase^18^. The fluidification effects of DHA, already observed by DSC measurements and by fluorescence spectroscopy, were thus confirmed. No domains were observed in the presence of DHA, indicating that the heterogeneity observed by DSC (Fig. 1) does not arise from large, i.e. micron-sized, domains. Instead, the presence of nanodomains with heterogeneous compositions, fatty acid-rich and -poor, could be proposed.

### A comparison with DHA in phospholipid form

**Figure 7:**
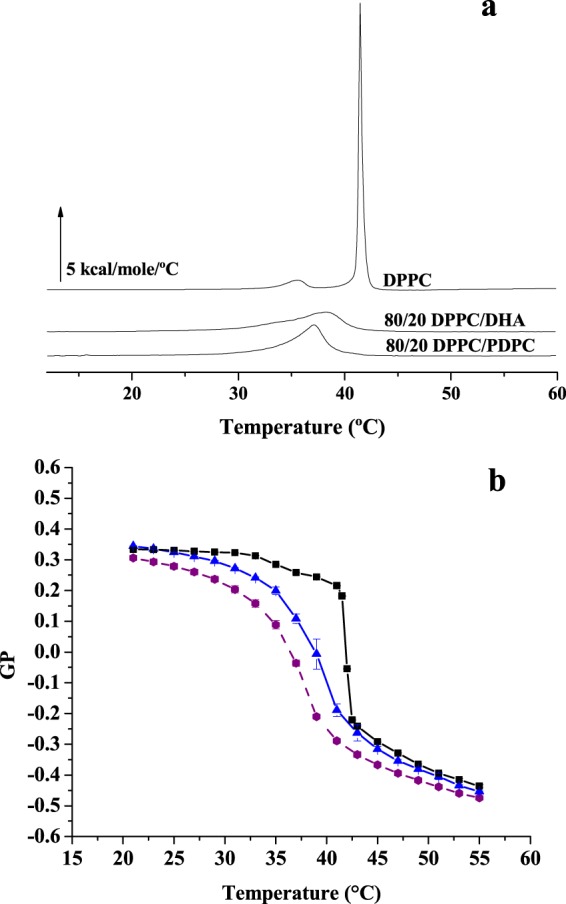
(**a**) Representative thermograms and (**b**) temperature scans of Laurdan GP value of a DPPC bilayer in the absence (black) or presence of 20 mol% DHA (blue) or of 20 mol% PDPC (purple). Data in (**b**) are average values ± S.D. (n = 3 independent liposome preparations). Sometimes the error bars are smaller than the symbols.

Some experiments were performed with DHA integrated in a phospholipid structure, namely 1-palmitoyl-2-docosahexanoyl phosphatidylcholine (PDPC). DSC thermograms of pure DPPC, and of DPPC containing 20 mol% of either DHA or PDPC are shown in Fig. 7a. In both cases the pre-transition had disappeared, the main transition had shifted to lower temperatures and it was broadened, and the enthalpy change ΔH was decreased. These are probably common features of all mixtures with DPPC with low-T melting components. In our particular case though, it should be noted that all these effects were slightly more marked for the PDPC than for the free fatty acid, suggesting that, of the two fluidifying lipids, PDPC is the one that mixes better with DPPC and perturbs less the bilayer structure. This conclusion is also supported by Laurdan data obtained from the same samples (Fig. 7b).

### Further control experiments

**Figure 8:**
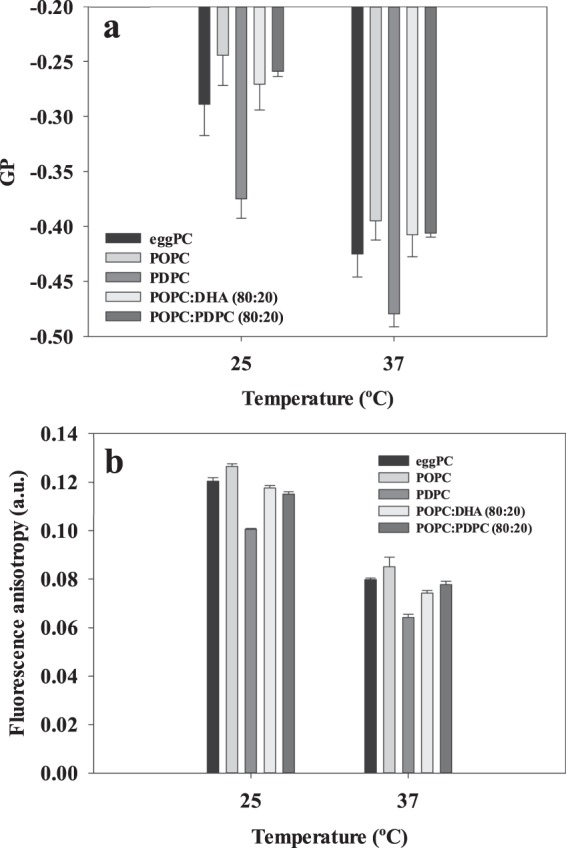
Effects of omega-3 fatty acids on the fluidity of different PC phospholipid bilayers. (**a**) Laurdan generalized polarization. (**b**) DPH anisotropy. Average values ± S.D. (n = 2 independent liposome preparations).

The very slight fluidifying effect of DHA or EPA on egg PC bilayers could be due to the fact that egg PC itself contains a non-negligible proportion of polyunsaturated fatty acids (typically over 20% di- or poly-unsaturated). To clarify this matter a series of experiments were performed in which DHA and PDPC were added to POPC bilayers, containing only fully saturated and monounsaturated fatty acids. Data of Laurdan GP at 25 °C and 37 °C are shown in Fig. 8a. GP values for egg PC and POPC are rather similar, and the effects of 20% DHA or PDPC on POPC bilayers are negligible from the point of view of fluidity as revealed by Laurdan GP measurements.

Another aspect to be considered is that Laurdan reports mainly on changes taking place near the lipid-water interface, while long-chain omega-3 fatty acids should be exerting their effects at the hydrophobic core. For this reason, the Laurdan experiments in Fig. 8a were repeated with DPH, a probe known to partition mainly with the fatty acyl chain region in the bilayer. In addition, the experiments by Ehringer *et al*.^9^, mentioned in the Introduction, were performed with DPH. Our DPH values are shown in Fig. 8b. Again 20 mol% DHA or PDPC hardly decrease DPH fluorescence anisotropy, i.e. increase fluidity in POPC bilayers.

## Discussion

Membrane fluidity is recognized as an essential property of cell membranes^19^. The main results in this paper concern the effects of omega-3 fatty acids on the fluidity of phosphatidylcholine bilayers and the miscibility of those free fatty acids with phospholipids. These two aspects deserve a separate discussion, together with the putative physiological implications of our study.

Fluidification effects have been studied in bilayers that were either in the gel or the fluid state at room temperature, respectively DPPC on one side, POPC, egg PC or egg SM on the other. With DPPC bilayers, inspection of the DSC thermograms (Figs 1 and 2) or of the Laurdan ‘GP *vs*. temperature’ plots (Figs 4 and 5a) does not reveal any effect of EPA or DPA that is substantially different from that of OA. The data in Table 1, from experiments with 20 mol% free fatty acid, indicate that the effects of EPA or DHA on melting temperatures, transition widths and enthalpy variations, respectively ΔT_m_, ΔT_1/2_, and ΔΔH, are 10–100% larger than those caused by OA, thus of the same order of magnitude for all three fatty acids, even if DPA and DHA contain 5 and 6 double bonds, respectively, and OA only 1. Even when the thermograms are decomposed into their Gaussian components (Fig. 1) the component endotherms and their thermodynamic parameters (Fig. 3) vary almost in parallel for the three fatty acids under investigation. This implies that previous suggestions that DHA has “unique” biophysical effects on membranes^13,20^ cannot be explained by its effects on membrane fluidity. Indeed, no indications of “hyperfluidizing” effects of EPA or DHA^13^ were found in our studies. With fluid bilayers, our Laurdan observations (Figs 5 and 8) of the small effects of OA, EPA or DHA on fluid bilayers (POPC, egg PC, egg SM, and their mixtures with cholesterol) are in good agreement with fluorescence polarization data from Ehringer *et al*.^9^. In summary, all three unsaturated fatty acids under study, OA, EPA and DHA exert some fluidizing effect that is quite clear for bilayers in the gel state (DPPC), much smaller for bilayers in the liquid ordered state (mixtures containing cholesterol), and rather negligible for bilayers in the fluid disordered state (egg PC, POPC). The effect of all three fatty acids is quite similar. It appears as if “hyperfluidity” claims for omega-3 fatty acids are supported neither by the current nor by past published results, to the authors’ knowledge.

**Table 1:** Effect of 20 mol% free fatty acid on the thermodynamic parameters of the main gel-fluid transition of DPPC. *Average values of two independent measurements*.

	ΔT_m_ (°C)	ΔT_1/2_ (°C)	ΔΔH (kcal/mole)
OA	2.80	3.60	1.30
EPA	3.12	5.36	2.30
DHA	3.30	5.91	2.65

DSC provides information that is difficult to obtain from fluorescence polarization data, namely the miscibility of bilayer components. In general, ideal miscibility should lead to symmetrical endotherms. In our case, addition of free fatty acids to DPPC bilayers leads to asymmetric signals in all cases, indicating non-ideal mixing, i.e. the coexistence of fatty-acid-rich and -poor regions in the bilayer. This is probably at the basis of the observations by Williams *et al*.^21^ that DHA induces a restructuring of lateral microdomains on the surface of living cells. This has been further investigated by the same authors in model membranes^22^ using NMR, with the result that phospholipids containing EPA or DHA partitioned preferentially into sphingomyelin- and cholesterol-rich domains. It is interesting in this respect that the non-ideal miscibility observed for unsaturated free fatty acids and DPPC (Fig. 1) is retained when the unsaturated fatty acid (at least EPA or DHA) are part of a phospholipid, as partially shown as well by our experiments with PDPC (Fig. 7a,b). Confocal fluorescence microscopy (Fig. 6) suggests that the fatty-acid-rich or -poor domains are in the nm range, i.e. the presumed range of ‘raft’ nanodomains^16,22^.

Cell membranes exist mostly, if not exclusively, in the fluid or liquid-crystalline state^19^. Both Ehringer *et al*.^9^ and ourselves (Fig. 5b) have shown that polyunsaturated omega-3 fatty acids do not modify the fluidity of membranes in the liquid-crystalline state, thus any physiological effects of these fatty acids are unlikely to occur through changes in membrane fluidity. Non-ideal miscibility in turn has been observed by us (Fig. 1) and other authors [see review in^19^], sometimes also in cell membranes, and could have observable consequences on the cell physiology.

Available data suggest that long-chain omega-3 fatty acids might exert their physiological roles in more subtle and/or specific ways than through changes in the overall fluidity of the bilayer. Gawrisch and co-workers^23,24^, using solid state NMR, have proposed that DHA-containing phospholipids cause a certain degree of membrane elastic stress that could modulate the function of integral receptor proteins such as rhodopsin. These authors observed as well the existence of weakly-specific interactions of DHA with spatially distinct sites of rhodopsin. Brockman *et al*.^8^ pointed out the electrostatic and lateral packing properties of *sn*-2-docosahexaenoyl chains as the origin of their peculiar interactions with proteins, beyond the mere changes in fluidity. More recently Kinnun *et al*.^25^ found that DHA modified the size and composition of sphingomyelin- and cholesterol-rich domains in mixtures with PDPC and POPC. Shaikh and co-workers^26,27^ have reported that omega-3 enhanced the formation of ordered microdomains in murine B cells, and that this was accompanied by effects on cell function. Thus there appears to be a whole series of physico-chemical properties that could explain the various physiological effects of omega-3 fatty acids without involving an increase in membrane fluidity as the main explanation.

An additional aspect in which long-chain polyunsaturated fatty acids could be relevant is the essential and widespread phenomenon of membrane fusion in cells. Ehringer *et al*.^9^ had observed that DHA facilitated vesicle-vesicle fusion much more than linolenic acid. Pinot *et al*.^28^ described how polyunsaturated phospholipids facilitate membrane deformation and fission promoted by endocytic proteins. Investigations in our laboratory^29,30^ also showed that arachidonic (C20:4) but not arachidic (C20:0) acid increased the fusion rate in a model system. In the latter study, the effect of the polyunsaturated acid was related to its intrinsic negative curvature, and consequently to its capacity to induce non-lamellar phase formation, confirmed by P^31^-NMR measurements^29,30^. The latter would be an as yet unexplored possible mechanism of action for omega-3 fatty acid physiological effects.

A final word concerning the physiological relevance of our observations. In many experiments described in this and other papers dealing with omega-3 fatty acids, these reagents are used at concentrations that are well above those found in cell membranes. However an important aspect may be overlooked here. When, under certain cellular conditions, a phospholipase A_2_ is activated, a transient and localized increase in free fatty acid will occur, and part of the physiological effects of that phospholipase activity might be due just to this temporary increase in fatty acid concentration. Our experiments with model membranes would then reflect those transiently perturbed bilayer regions that can be the locus of a given omega-3 effect, be it modulation of intrinsic protein activity, or membrane fusion, or other. However the data in Fig. 7 suggest that omega-3 fatty acids exert a similar effect on fluidity either in free form or making part of a phospholipid, thus phospholipase A_2_ activation should not cause major omega-3-induced local changes in membrane fluidity.

## Materials and Methods

### Materials

Chloroform, dichloromethane and methanol, HPLC-grade solvents, were obtained from Merck (Darmstadt, Germany). Egg phosphatidylcholine (PC), egg sphingomyelin (SM), 1,2-dipalmitoyl-*sn*-glycero-3-phosphatidylcholine (DPPC) and 1-palmitoyl-2-docosahexaenoyl-*sn*-glycero-3-phosphocholine (PDPC) were obtained from Avanti Polar Lipids (Birmingham, AL, USA). Diphenyl hexatriene (DPH) was supplied by Sigma-Aldrich (Madrid, Spain). Docosahexaenoic acid, eicosapentaenoic acid and oleic acid were provided by Cayman Chemical (Ann Arbor, MI).

### Sample preparation

Lipids were mixed in chloroform at the appropriate ratios, and solvent was evaporated under nitrogen. Then the dried lipids were left for at least 2 h under vacuum to remove solvent traces. For MLV preparation the lipids were hydrated in 20 mM PIPES, 150 mM NaCl, 1 mM EDTA, pH 7.4 buffer, followed by vigorous vortex mixing. Sample and buffer were maintained at a temperature above the lipid main transition temperature, so that the lipids were in the fluid state throughout the procedure. For spectrofluorometric studies samples were additionally sonicated in a bath sonicator, also above the lipid main transition temperature, for 10 min, to ensure homogeneity. Precautions were taken throughout the procedure to minimize oxidation^9^, including limiting exposure to light and using an environment purged with nitrogen during manipulations. In some cases butyl hydroxyl toluene (BHT) was added to the lipid mixtures in chloroform, at a 55:1 PUFA:BHT mol ratio, but no clear differences were observed with the BHT-free samples.

Giant Unilamellar Vesicles (GUVs) were prepared using the electroformation method. For vesicle observation, a home-made chamber was used that allows direct GUV visualization under the microscope^31^. Stock lipid solutions (0.2 mg/mL total lipid containing 0.2 mol % lissamine rhodamine PE (Rho-PE)) were prepared in a chloroform/methanol (2:1, v/v) solution. Then 3 μL of the appropriate lipid stocks were added onto the surface of Pt electrodes and solvent traces were removed by placing the chamber under high vacuum for at least 2 h.

The Pt electrodes were covered with 400 µL Millipore filtered Milli-Q water previously equilibrated at 75 °C. The Pt wires were connected to an electric-wave generator (TG330 function generator, Thurlby Thandar Instruments, Huntington, United Kingdom) under AC field conditions (10 Hz, 0.9 V) for 2 h at 75 °C. The generator and the water bath were switched off, and vesicles were left to equilibrate at room temperature for 1 h.

### Differential Scanning Calorimetry (DSC)

All measurements were performed in a VP-DSC high-sensitivity scanning microcalorimeter (MicroCal, Northampton, MA). Both lipid and buffer solutions were fully degassed before loading into the appropriate cell. Buffer was 20 mM PIPES, 150 mM NaCl, 1 mM EDTA, pH 7.4. Lipid suspensions were loaded into the microcalorimeter in the form of multilamellar vesicles prepared as described above. A final amount of 0.5 ml at 0.5 mM total lipid concentration was loaded into the calorimeter, and up to six heating scans were performed at 45 °C/h between 10 and 60 °C for all samples. Lipid concentration was determined as lipid phosphorus, and used together with data from the last scan, to obtain normalized thermograms. The software Origin 7.0 (MicroCal), provided with the calorimeter, was used to correct the thermogram baselines and to determine the different parameters for the scans. Transition temperatures T_m_ were measured at the peak maximum, widths at half-height (T1/2) refer to widths of the overall recorded endotherm, and enthalpies were computed from the area under the endotherm peak. The same parameters were measured for the individual endotherm components when required.

### Fluorescence spectroscopy

The emission maxima of Laurdan in phospholipid bilayers depend upon the phase state of the phospholipids, being blue in the gel (maximum emission = 440 nm) and green in the liquid crystalline phase (maximum emission = 490 nm). This shift of the emission spectrum has been attributed to dipolar relaxation processes occurring in the phospholipid liquid–crystalline phase but not in the gel phase^15^. The generalized polarization (GP) of Laurdan was measured in an Aminco Bowman Series 2 spectrofluorometer (ThermoFisher Scientific, Waltham, MA) equipped with thermoregulated cell holders. GP was calculated with equation (1):

1 $${\boldsymbol{G}}{\boldsymbol{P}}=\frac{{{\boldsymbol{I}}}_{{\bf{440}}}-{{\boldsymbol{I}}}_{{\bf{490}}}}{{{\boldsymbol{I}}}_{{\bf{440}}}+{{\boldsymbol{I}}}_{{\bf{490}}}}$$

where *I*_440_ and *I*_490_ are the emission intensities at 440 and 490 nm, respectively, when exciting at 360 nm. The final probe/lipid molar ratio was 1/500.

### DPH fluorescence anisotropy

The appropriate lipids were mixed with DPH in organic solvent at a 1:200 DPH:lipid mol ratio. MLV were formed in buffer as indicated above. The samples were left to equilibrate at the temperature of the experiment for 1 h. Steady-state fluorescence.

Anisotropy measurements were performed on an Aminco Bowman Series 2 spectrofluorometer (ThermoFisher Scientific, Waltham, MA) equipped with excitation and emission polarizers. Excitation and emission wavelengths were set at 360 and 454 nm, respectively. Fluorescence anisotropy (r) was calculated using equation (2)

2 $$r=({I}_{VV}-G{I}_{VH})/({I}_{VV}+2G{I}_{V{\rm{H}}})$$

where I_VV_ and I_VH_ are the fluorescence intensities determined at vertical and horizontal orientations of the emission polarizer, respectively, when the excitation polarizer is set in the vertical position. The G factor, which compensates for differences in detection efficiency for vertically and horizontally polarized light, was calculated from the fluorescence intensity ratio of vertical and horizontal emissions when the excitation polarizer is set in the horizontal position (I_HV_/I_HH_).

### Confocal microscopy of GUVs

After GUV formation, the chamber was placed onto an inverted confocal fluorescence microscope (Nikon D-ECLIPSE C1, Nikon, Melville, NY). The excitation wavelength for Rho-PE was 561 nm, and the images were collected using a band-pass filter of 593 ± 20 nm. Image treatment and quantitation were performed using the software EZ-C1 3.20 (Nikon).
